# Chitosan-Based Nanoparticles and Biomaterials for Pulp Capping and Regeneration: A Systematic Review with Quantitative and Evidence-Mapping Synthesis

**DOI:** 10.3390/biomimetics10120822

**Published:** 2025-12-09

**Authors:** Saleh Ali Alqahtani, Mohammad Alamri, Ghadeer Alwadai, Naif N. Abogazalah, Vinod Babu Mathew, Betsy Joseph

**Affiliations:** 1 Department of Restorative Dental Sciences, College of Dentistry, King Khalid University, Abha 61421, Saudi Arabia; ssofan@kku.edu.sa (S.A.A.); moabalamri@kku.edu.sa (M.A.); gsalwadai@kku.edu.sa (G.A.); vbmathew@kku.edu.sa (V.B.M.); 2 Department of Restorative Dental Sciences, College of Dentistry, King Faisal University, Al Ahsa 31982, Saudi Arabia; 3 Department of Periodontics, Saveetha Institute of Medical and Technical Sciences, Chennai 600077, India; 4 Department of Oral and Maxillofacial Diseases, Faculty of Medicine, University of Helsinki, 00014 Helsinki, Finland

**Keywords:** chitosan, nanoparticles, pulp capping, regenerative endodontics, odontogenic differentiation, evidence mapping, GRADE analysis

## Abstract

Preserving dental pulp vitality is a key goal in minimally invasive dentistry. Conventional materials such as calcium hydroxide and mineral trioxide aggregate (MTA) are effective but limited in bioactivity and mechanical strength. This systematic review evaluated the biological efficacy of chitosan-based nanoparticles and biomaterials for pulp capping and regeneration. Following PRISMA 2020 guidelines, electronic searches were conducted across five databases up to April 2025. Controlled in vitro and animal studies using chitosan-based nanoparticles, hydrogels, or composite scaffolds were included. Risk of bias was assessed using SYRCLE (animal) and ToxRTool (in vitro), and certainty of evidence was rated via the GRADE-Preclinical framework. Due to methodological heterogeneity, data were synthesized using direction-of-effect coding and visualized through Albatross and heatmap plots. Sixteen studies met the criteria, consistently demonstrating enhanced cell viability, mineralization, and upregulation of odontogenic and angiogenic markers (BMP-2, TGF-β_1_, VEGF, DSPP) compared with MTA or calcium hydroxide. Animal models confirmed improved angiogenesis, reparative dentin formation, and pulp vitality preservation. Despite uniformly positive biological outcomes, the overall certainty was rated Low to Very Low owing to small samples and unclear randomization. Chitosan-based biomaterials show promising regenerative potential, warranting well-designed preclinical and clinical studies for translational validation.

## 1. Introduction

Preservation and regeneration of the dental pulp have become central objectives in contemporary restorative and regenerative dentistry. Traditional pulp-capping materials such as calcium hydroxide and mineral trioxide aggregate (MTA) have demonstrated clinical success in promoting reparative dentin formation; however, comparative preclinical reports increasingly test bioactive alternatives that may outperform or complement these standards in key biological readouts, including mineralized barrier formation and tissue preservation [1,2]. In particular, some chitosan-containing systems have shown superior dentin bridge quality or pulp preservation relative to MTA in animal or cell models, motivating systematic evaluation of these strategies [1,3].

Chitosan, a naturally derived polysaccharide obtained from the deacetylation of chitin, has emerged as a promising biofunctional polymer in dental tissue engineering. Across in vitro and in vivo models, chitosan-based constructs are frequently biocompatible and non-cytotoxic, support cell adhesion, and can exhibit antimicrobial or anti-inflammatory behavior with properties desirable for pulp healing [4,5,6,7,8]. These features make chitosan an attractive scaffold component and carrier for bioactive payloads in pulp capping and regenerative procedures [1,9].

Recent advances in nanotechnology and biomaterials engineering have yielded diverse chitosan formats, such as nanoparticles, hydrogels, and composite scaffolds, enabling controlled growth-factor delivery, tuned porosity/architecture, and reinforced mechanics, as well as incorporation of functional co-components such as nano-hydroxyapatite, bioactive glass, and metal/oxide nanoparticles [1,3,4,7,10,11,12]. Examples include VEGF-releasing chitosan/β-glycerophosphate hydrogels that sustain factor delivery and enhance odontogenic differentiation, platelet-derived adjuncts that boost mineralization on chitosan-composite scaffolds, extracellular matrix-loaded chitosan/alginate hydrogels that stimulate pulp-cell activity, and silver-doped bioactive glass/chitosan systems that couple reparative and anti-inflammatory effects [3,7,10,12].

Preclinical evidence from both in vitro and animal studies suggests that chitosan-based formulations can promote odontoblastic differentiation and mineralized matrix deposition, and can upregulate key mediators relevant to pulp healing, such as TGF-β1, BMP-2, and VEGF, while supporting angiogenesis and reparative dentin formation [9,13,14,15]. Nevertheless, findings across studies remain heterogeneous due to variations in chitosan chemistry and format, co-components (e.g., HA, PRP/FG, TiO_2_, Ag), cell sources, assay systems, and experimental conditions [1,4,11,16]. While numerous reports indicate promising outcomes including improved cell viability/adhesion, enhanced odontogenic marker expression, and favorable dentin bridge characteristics, there has been no integrated quantitative synthesis across these datasets to determine overall effect sizes and consistency [5,10,12].

Therefore, this systematic review aimed to comprehensively evaluate the biological efficacy of chitosan-based nanoparticles and biomaterials in pulp capping and regeneration, focusing on both in vitro and in vivo preclinical evidence. Specifically, we sought to determine whether chitosan-based formulations improve cell viability, odontogenic differentiation, angiogenesis, and reparative dentinogenesis compared with conventional agents such as MTA and calcium hydroxide, and to use evidence mapping and quantitative synthesis to critically appraise data quality and identify translational gaps for future research and clinical application.

## 2. Materials and Methods

### 2.1. Protocol and Registration

This systematic review followed the PRISMA 2020 guidelines and was registered on Open Science Framework. The review question, eligibility criteria, and analytic framework were prospectively defined. The review adhered to the PICO model to ensure a transparent and replicable design.

### 2.2. Research Question

Does the use of chitosan-based nanoparticles or biomaterials improve reparative dentin formation and pulp healing more effectively than conventional pulp capping agents?

### 2.3. Objectives

1. To systematically identify and appraise preclinical (in vitro and animal) studies evaluating chitosan-based nanoparticles or biomaterials for pulp capping or regeneration.
2. To quantitatively synthesize comparable outcomes using meta-analysis where feasible, and to use direction-of-effect synthesis and evidence mapping where meta-analysis is not appropriate.

### 2.4. Eligibility Criteria

*Inclusion* 

- Controlled in vitro or in vivo studies evaluating chitosan-based nanoparticles or biomaterials (scaffolds, hydrogels, composites) for dental pulp capping or regeneration.
- Studies reporting quantitative or digitizable outcomes (mean ± SD/SE, *p*-values).
- Comparators: calcium hydroxide, MTA, Biodentine, or other pulp-capping materials.

*Exclusion* 

- Reviews, editorials, letters, or conference abstracts.
- Material characterization studies without biological outcomes.
- Case reports, single-arm experiments, or studies lacking control groups.

### 2.5. PICO Framework

Population (P): Human, animal, or in vitro pulp/pulp stem cell models; Intervention (I): Chitosan nanoparticles or chitosan-based biomaterials (hydrogels, scaffolds, composites); Comparator (C): Conventional materials (MTA, calcium hydroxide, Biodentine), or no treatment and Outcomes (O): Reparative dentin formation, pulp healing, cell proliferation, odontogenic differentiation (e.g., BMP-2, TGF-β1, DSPP), angiogenesis (VEGF), and biocompatibility (cell viability assays).

### 2.6. Search Strategy

A comprehensive literature search was performed in PubMed/MEDLINE, Scopus, Embase, Web of Science, Cochrane CENTRAL, and Google Scholar.

Search strings included MeSH and free-text terms such as:

(“chitosan” OR “chitosan nanoparticle*” OR “chitosan hydrogel” OR “carboxymethyl chitosan”)

AND (“pulp capping” OR “pulp regeneration” OR “dentin bridge” OR “dental pulp stem cell*”)

No language or date restrictions were applied. Reference lists of included studies and related reviews were hand-searched to ensure completeness.

### 2.7. Study Selection

**Figure 1:**
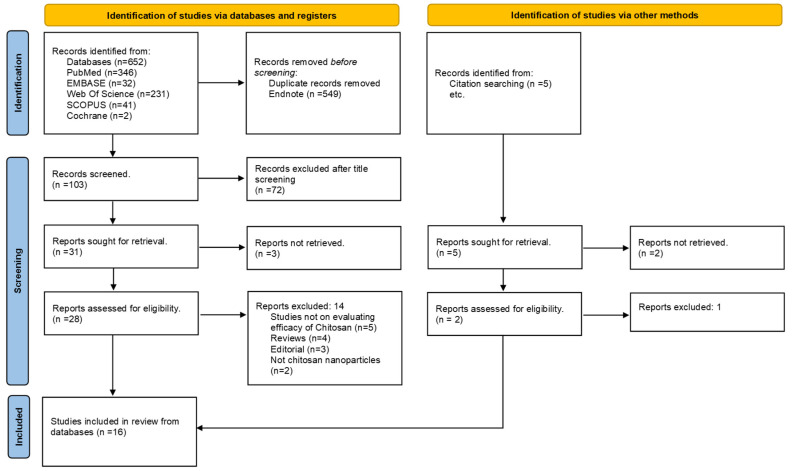
PRISMA Flowchart.

All retrieved references were imported into EndNote 20 for de-duplication. Two reviewers independently screened titles and abstracts, followed by full-text eligibility assessment. Disagreements were resolved through discussion or consultation with a third reviewer. Exclusion reasons at the full-text stage were documented and presented in a PRISMA flow diagram (Figure 1).

### 2.8. Data Extraction and Management

Data were independently extracted by two reviewers into a pre-structured Excel sheet. Extracted fields included study characteristics (author, year, study type, model or cell line, sample size, intervention type, comparator, outcomes, and timepoints), quantitative results (mean, SD/SE, *p*-values), and key methodological notes.

When numerical results were reported only graphically, data were digitized using WebPlotDigitizer v4.6 (Automeris, USA) after calibrated y-axis scaling. Extracted values were verified against axis grids and replicate means. For studies reporting SE, SD values were derived using SD = SE × √n.

All extracted and derived data were verified and documented in a digitization log for reproducibility.

### 2.9. Risk of Bias Assessment

#### 2.9.1. In Vivo Studies

Animal studies were appraised using the SYRCLE Risk of Bias tool [17], evaluating ten domains: random sequence generation, baseline comparability, allocation concealment, random housing, caregiver and assessor blinding, incomplete outcome data, selective reporting, and other biases. Each domain was rated Low, Unclear, or High risk.

#### 2.9.2. In Vitro Studies

Since there is no universally accepted RoB framework for in vitro research, an adapted composite checklist (ToxRTool + JBI) was employed to ensure structured appraisal [18]. In vitro experiments contributing quantitative data (*n* = 5) were evaluated.

Domains included: test system description, control adequacy, replication, randomization of treatment, blinding of measurement, assay validation, data completeness, outcome reporting, statistical analysis, and funding/conflict transparency. Each domain was scored as Low (L), Unclear (U), or High (H), and total scores (maximum = 20) were classified as Low (≥16), Moderate (10–15), or High (<10) risk.

While randomization and blinding are rarely feasible in vitro, these domains were retained to assess transparency and rigor. Automated assays (e.g., MTT, CCK-8, ELISA) were considered objective for blinding.

### 2.10. Quantitative and Semi-Quantitative Synthesis

#### 2.10.1. Meta-Analysis Feasibility and Approach

A formal meta-analysis was planned for outcomes with homogeneous quantitative measures (e.g., cell viability [MTT/CCK-8], TGF-β_1_, BMP-2). However, due to differences in assay type, timepoints, and incomplete variance reporting, a full pooled meta-analysis was not feasible. Instead, quantitative trends were summarized descriptively using extracted mean ± SD values and direction-of-effect synthesis. Where ≥2 comparable studies were available, forest-style plots and evidence-weighted visualizations (bubble and heatmaps) were produced to depict the magnitude and consistency of effects.

#### 2.10.2. Direction-of-Effect and Weighting

When complete variance data were unavailable, direction-of-effect synthesis was applied [19]. Each study’s outcome was coded as: +1 = positive, 0 = neutral, −1 = negative, weighted by completeness:

- mean ± SD (weight = 2)
- *p*-value only (weight = 1)
- qualitative (weight = 0.5)

#### 2.10.3. Certainty of Evidence

Certainty for in vivo outcomes (e.g., reparative dentin, TGF-β_1_, BMP-2, VEGF) was assessed using the GRADE-Preclinical framework [20], which adapts standard GRADE domains (risk of bias, inconsistency, indirectness, imprecision, and publication bias) for animal and in vitro research. Each outcome was rated as High, Moderate, Low, or Very Low certainty based on the overall confidence in the body of evidence.

## 3. Results

### 3.1. Overview

A total of 16 studies (11 in vitro and 5 in vivo) published between 2018 and 2025 met the inclusion criteria after screening 19 eligible records. Three studies [2,6,21] were excluded at the data-harmonization stage as they lacked pulp or pulp-cell-related regenerative outcomes. The PRISMA flow diagram summarizing the selection process is shown in Figure 1 while Table 1 shows study characteristics of included studies (n = 16).

#### 3.1.1. In Vivo Findings

Five animal studies reinforced the regenerative potential of chitosan formulations through histological and biochemical outcomes. Widyastuti et al. demonstrated that nanochitosan derived from red snapper scales markedly improved TGF-β_1_ and reparative dentin formation, while their subsequent work confirmed significant BMP-2 and TGF-β_1_ upregulation compared with calcium hydroxide [15]. Sularsih et al. reported that chitosan–hydroxyapatite composites enhanced VEGF expression, vascularization, and fibroblast proliferation in rat pulp tissue, outperforming conventional agents [14]. Hoveizi et al. found that chitosan hydrogels containing TiO_2_ nanoparticles and human endometrial stem cells achieved the highest dentin formation quality and quantity, suggesting synergistic cell–material interactions [11]. Similarly, Zhu et al. demonstrated that silver-doped chitosan hydrogels yielded superior pulp preservation and reparative dentinogenesis compared with MTA, likely via MAPK pathway activation [3]. Taken together, in vivo findings indicate that chitosan-based biomaterials foster angiogenesis, odontogenesis, and inflammation modulation, leading to improved dentin bridge formation and pulp healing compared with traditional materials.

#### 3.1.2. In Vitro Findings

Across eleven in vitro studies, chitosan-based nanoparticles, scaffolds, and composites consistently enhanced cell viability, proliferation, and odontogenic differentiation in pulp-derived stem cells compared with conventional or untreated controls.

Formulations such as chitosan–bioceramic composites [5], nHA–chitosan–gelatin–alginate scaffolds with PRF [23], and nano phosphorylated pullulan–carboxymethyl chitosan scaffolds [16] demonstrated improved cytocompatibility and bioactivity. Hydrogels enriched with extracellular matrix [2] and nHA–carboxymethyl chitosan composites [13] showed increased ALP, ARS, OPN, and DSPP expression, reflecting enhanced mineralization. Similarly, PCL–nanochitosan–hydroxyapatite scaffolds [1] and chitosan–gelatin–nHA + PRP–FG composites [7] induced upregulation of BMP2 and RUNX2, while polyhydroxybutyrate–chitosan–nano-bioglass scaffolds [4] significantly increased SHED cell viability. Further, vitamin D_3_–loaded chitosan–calcium–aluminate scaffolds and chitosan/β-glycerophosphate hydrogels [12] enhanced odontoblastic differentiation and VEGF-mediated proliferation, respectively. Cobalt-incorporated chitosan scaffolds promoted cell adhesion and proliferation without cytotoxicity, confirming favorable biological compatibility [8]. Overall, in vitro evidence supports that chitosan-based materials are biocompatible, mineralization-promoting, and conducive to pulp-dentin tissue regeneration.

#### 3.1.3. Integrated Summary

The convergence of in vitro and in vivo data highlights chitosan’s dual role as a biocompatible matrix and biological modulator, stimulating both cellular differentiation and growth factor expression (BMP-2, TGF-β_1_, VEGF). Although methodological heterogeneity limited full meta-analytic pooling, direction-of-effect synthesis and evidence mapping confirmed uniformly positive trends across studies, underscoring the translational promise of chitosan-based biomaterials for regenerative endodontics.

### 3.2. Risk of Bias

#### 3.2.1. In Vivo Studies (SYRCLE Assessment)

**Figure 2:**
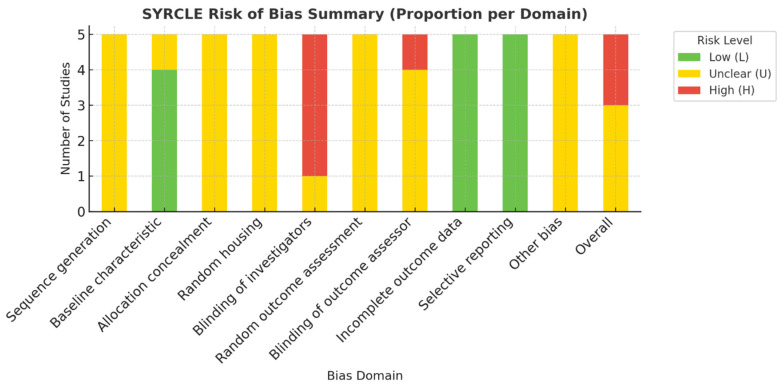
Visual Summary of SYRCLE Risk of Bias Across In Vivo Studies [17].

Across the five animal studies, sequence generation and allocation concealment were rarely reported, and random housing was inconsistently applied. Outcome assessor blinding was unclear in all but one study. Selective reporting and incomplete data were generally low risk. Overall, three studies showed moderate [11,14,15], and two studies high [3,22], risk of bias. Results are summarized in Table 2 and Figure 2.

#### 3.2.2. In Vitro Studies (ToxRTool/JBI Assessment)

**Figure 3:**
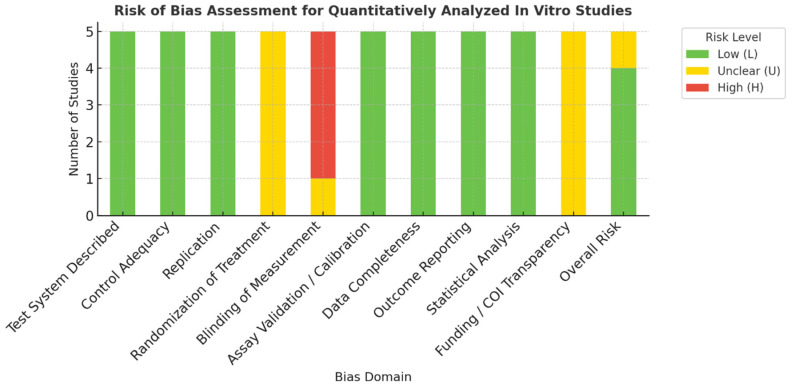
Summary Visualization of Risk of Bias Across Quantitatively Analyzed In Vitro Studies [18].

All included in vitro studies adequately described their test systems and used validated assays (MTT, CCK-8, ALP, ELISA, OPN). Appropriate controls and triplicate replicates were consistently used; however, randomization and blinding were unreported in most studies. All reported complete datasets with valid statistical analyses (typically ANOVA). Funding and conflict disclosures were inconsistently stated. Overall, most studies were rated as low to moderate risk of bias. (Table 3 and Figure 3).

### 3.3. Quantitative Findings

#### 3.3.1. Meta-Analysis Feasibility

Pooled meta-analysis was planned for homogeneous outcomes such as cell viability (MTT/CCK-8) and growth factor expression (TGF-β_1_, BMP-2). However, due to variability in assay type, timepoints, and incomplete variance data, a full pooled analysis was not feasible.

#### 3.3.2. Direction-of-Effect Synthesis

**Figure 4:**
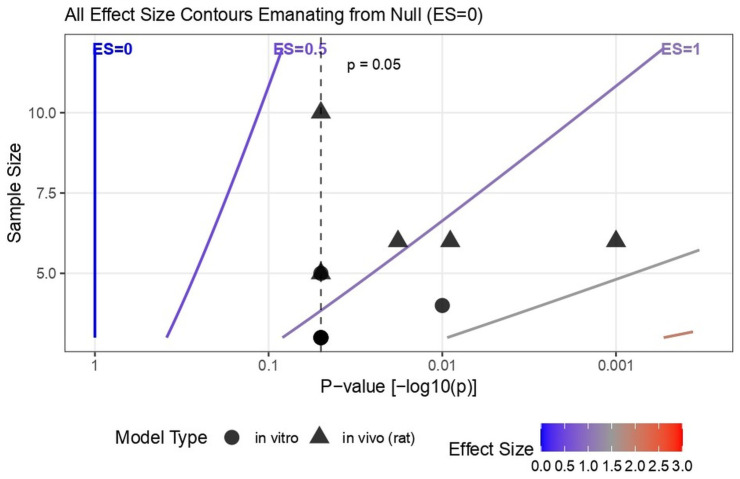
Direction-of-Effect Synthesis shown using the Albatross Plot.

Due to heterogeneity in assay types, reporting formats, and incomplete variance data, direction-of-effect synthesis was used to summarize trends across in vitro and in vivo studies. The Albatross plot [24] (Figure 4) showed that most data points were positioned to the right of the null axis and below the *p* = 0.05 contour lines, indicating statistically significant positive effects of chitosan-based biomaterials. Both in vitro and in vivo outcomes clustered in this region, suggesting generally improved regenerative responses across models. The Bubble plot [25] (Figure S1) mapped evidence distribution by outcome domain, with larger and darker bubbles concentrated in in vitro categories such as cell viability, mineralization, and differentiation, while in vivo outcomes for angiogenesis and reparative dentin formation also demonstrated positive trends. The Heatmap [19] (Figure S2) reinforced these findings, with dense red gradients across nearly all domains reflecting the predominance of positive (+1) directional codes and higher evidence weights. Together, these visualizations confirm that, despite methodological heterogeneity, the overall direction of evidence supports the beneficial biological performance of chitosan-based nanoparticles and biomaterials in pulp capping and regeneration. Table 4 shows the direction-of-effect coding and weighting log.

### 3.4. Certainty of Evidence (GRADE-Preclinical Assessment) [20] 

Based on GRADE-Preclinical evaluation, the certainty of evidence supporting chitosan-based nanoparticles and biomaterials for pulp capping and regeneration ranged from Low to Very Low (Table 5). Downgrades were primarily due to methodological limitations (unclear randomization and blinding), small animal sample sizes, and indirectness to human clinical outcomes. Although all included studies reported biologically favorable effects, the confidence in these findings remains limited because the evidence is derived from preclinical models using surrogate outcomes that are far removed from direct clinical settings. Consequently, while chitosan-based formulations appear promising, their translational applicability to human dental practice cannot yet be confirmed and requires validation through well-designed clinical trials.

## 4. Discussion

This systematic review synthesized current in vitro and in vivo evidence on the biological efficacy of chitosan-based nanoparticles and biomaterials for dental pulp capping and regeneration. Across studies, chitosan formulations consistently enhanced cell viability, odontogenic differentiation, angiogenic signaling, and reparative dentin formation compared with conventional agents such as calcium hydroxide and mineral trioxide aggregate. Although methodological heterogeneity precluded a formal meta-analysis, the integrated use of direction-of-effect synthesis, Albatross plots, and evidence heatmaps revealed a coherent positive trend across multiple biological domains.

Chitosan, a partially deacetylated derivative of chitin, exhibits biocompatibility, antibacterial activity, and intrinsic biofunctionality. Its cationic amino groups electrostatically interact with negatively charged cell membranes, proteins, and extracellular matrix components, facilitating cellular adhesion and growth factor retention [6]. The reviewed studies indicate that these interactions may underlie enhanced odontoblastic differentiation and angiogenesis through upregulation of *TGF-β*_1_, *BMP-2*, and *VEGF* expression [7,26,27]. Widyastuti et al. demonstrated significantly higher *BMP-2* and *TGF-β*_1_ levels in a rat pulpitis model treated with nanochitosan compared with calcium hydroxide, corroborating prior evidence that chitosan scaffolds activate odontogenic signaling cascades [15].

Compositional modifications further strengthened chitosan’s regenerative profile. The addition of nano-hydroxyapatite [13,14] and PRP/Fibrin Glue [7] increased osteogenic marker expression (RUNX2, OPN, DSPP), while silver or TiO_2_ incorporation [3,11] introduced antimicrobial and angiogenic effects. These synergistic modifications mirror principles of biomimetic tissue engineering, where controlled ionic exchange, scaffold porosity, and biochemical signalling combine to replicate natural pulp microenvironments [3]. Most formulations sustained cell viability above 80% and demonstrated anti-inflammatory benefits, supporting the hypothesis that chitosan’s biological neutrality and active surface interaction collectively enhance pulp regeneration.

Among the various formulations, chitosan nanoparticles, chitosan-based hydrogels, and composites incorporating bioactive agents (calcium silicate or growth factors) appear most promising for clinical translation [19,20]. These modifications enhance biocompatibility, antibacterial effects, and controlled release properties, supporting more predictable pulp healing. However, further standardized preclinical and clinical studies are required. Using surrogate endpoints, such as dentin bridge thickness or early cellular responses, limits the ability to assess true pulp regeneration. These measures may overestimate treatment success because they do not capture long-term pulp vitality, functional vascularization, or innervation. Therefore, surrogate outcomes provide only partial insight into clinical regenerative potential.

Despite the absence of pooled effect estimates, alternative synthesis tools provided valuable insight. However, the Albatross plot was used only as a qualitative visualization to depict directional agreement across heterogeneous datasets. Because the contours are based on approximated combinations of *p*-values and sample sizes, the plot does not imply increased certainty or provide quantitative estimates of effect size. Its purpose is descriptive rather than confirmatory. Furthermore, in vitro and in vivo outcomes were not compared directly, as their biological complexity and endpoints differ substantially. The phrase ‘generally improved regenerative responses’ in the result section refers only to consistent positive trends observed within each model system. The Albatross plot integrates these findings to illustrate coherence in effect direction, not to equate biological outcomes or imply comparability across model hierarchies.

Complementary heatmap visualizations revealed dense positive gradients for odontogenic (*BMP-2*, *DSPP*), angiogenic (*VEGF*), and cellular (*MTT*, *CCK-8*) domains. The bubble plot further highlighted evidence density in cell viability and differentiation categories. These visual approaches, consistent with Cochrane recommendations for heterogeneous preclinical data [19,28], collectively indicate consistent biological enhancement despite methodological variability.

Among the five included in vivo studies, two implemented randomizations in allocation [11,14], reflecting early efforts toward experimental rigor. Nanochitosan and chitosan–hydroxyapatite composites notably improved dentin bridge integrity, angiogenesis, and *TGF-β*_1_ expression compared with MTA or Ca (OH)_2_. However, no human randomized controlled trials have yet validated these findings. Translation from preclinical success to clinical practice requires multicentric RCTs employing standardized protocols for randomization, histologic evaluation, and long-term pulp vitality assessment. Comparative studies involving current bioactive cements (e.g., Biodentine, Activa Bioactive) could further clarify relative performance and safety.

The GRADE-Preclinical appraisal rated overall certainty as Low to Very Low, consistent with limitations typical of biomaterials research. Downgrades arose from incomplete reporting of randomization and blinding, small animal sample sizes, and reliance on surrogate endpoints rather than clinically validated outcomes. Nonetheless, consistency in the direction of effects across independent laboratories supports biological plausibility. The GRADE framework contextualizes confidence rather than invalidating findings, it identifies domains requiring methodological reinforcement [20]. Future studies could strengthen certainty by adopting CONSORT-adapted reporting for animal experiments, pre-registered protocols, and harmonized biomaterial characterization standards. However, the limited number of eligible studies reduces the reliability of these assessments. With few studies, key GRADE domains such as inconsistency, imprecision, and publication bias cannot be evaluated with adequate statistical power. Additionally, methodological heterogeneity and variability in outcome reporting across preclinical studies further limit the confidence that can be placed in the overall certainty ratings. Therefore, the GRADE conclusions presented here should be interpreted cautiously and considered indicative rather than definitive.

Large-scale use of biomimetic and nano-engineered materials raises several regulatory and safety considerations. Their complex compositions and biological interactions may lead to cytotoxicity, immunogenicity, or unpredictable long-term degradation profiles. Moreover, these materials often fall outside conventional device or drug classifications, requiring extensive preclinical validation and adherence to evolving ISO and FDA guidelines. Ensuring consistent manufacturing quality, sterility, and stability remains an additional challenge. These factors underscore the need for robust regulatory evaluation before widespread clinical adoption

Publication bias is a plausible concern, as nearly all included studies reported positive findings. Negative or neutral results may remain unpublished, potentially inflating apparent efficacy. Additionally, in vitro assays cannot fully reproduce the dynamic, vascularized pulp environment, and few in vivo models evaluated long-term biodegradation or mechanical durability, key factors for clinical translation. Addressing these evidence gaps through standardized animal models and early-phase human trials will be essential to confirm reproducibility and safety.

Current computational and modeling tools also present limitations that restrict progress in predicting pulp tissue responses to chitosan-based biomaterials. Most existing platforms rely on simplified, static assumptions and are unable to replicate the complex biological microenvironment of the dental pulp, including inflammatory dynamics, vascular changes, and multi-cellular interactions. Furthermore, the lack of standardized input parameters and limited integration of molecular, cellular, and tissue-level data reduce the predictive accuracy of these models. These constraints highlight the need for more advanced, biologically informed computational frameworks to support the development and optimization of next-generation regenerative materials.

### Strength and Limitations

This review offers several notable strengths. This review provides an up-to-date and integrative overview of current evidence on chitosan-based nano-biomaterials for pulp capping and regeneration, synthesizing available findings from cellular and animal studies. Integration of alternative quantitative visualization tools (direction-of-effect coding, Albatross, and heatmaps) enhances interpretability where traditional meta-analysis was infeasible. Adherence to PRISMA 2020 standards, use of structured risk-of-bias tools (SYRCLE and ToxRTool), and incorporation of the GRADE-Preclinical framework collectively elevate methodological transparency. Furthermore, this synthesis identifies recurring mechanistic themes—*TGF-β_1_/BMP-2/VEGF* upregulation and enhanced mineralization—that bridge molecular and tissue-level outcomes, supporting translational coherence.

Nonetheless, certain limitations must be acknowledged. Considerable heterogeneity existed in chitosan chemistry (degree of deacetylation, molecular weight), nanoparticle size, co-component ratios, and experimental endpoints. Small sample sizes, short follow-ups, and inconsistent blinding contribute to potential bias. Publication bias and selective reporting cannot be excluded. Finally, the lack of quantitative pooling limits effect-size estimation, and the absence of human RCTs restricts clinical generalizability. Translating preclinical findings into clinical practice remains challenging because in vitro and animal models cannot fully replicate human pulp biology, patient variability, or real clinical conditions. Differences in inflammation, defect size, and application techniques, along with heterogeneous study protocols, limit the direct applicability of these results to clinical scenarios. Most studies lacked long-term follow-up, which limits confidence in the durability of the observed regenerative outcomes. Short observation periods may overestimate early healing and fail to capture long-term pulp vitality, material stability, or late complications. This gap highlights the need for studies with extended follow-up to validate these findings.

## 5. Conclusions

Within the limitations of the current evidence, chitosan-based nanoparticles and biomaterials demonstrate consistent biological advantages over conventional pulp-capping agents, enhancing odontogenic differentiation, angiogenesis, and reparative dentinogenesis. Visual synthesis approaches confirmed a strong positive directional trend across diverse preclinical models. However, the low overall certainty highlights that these findings should be interpreted *as biologically promising but not yet clinically validated*. The translational potential of chitosan lies in its biocompatibility, tunable physicochemical properties, and versatility as a carrier for bioactive molecules. Future multicentric randomized studies—incorporating standardized protocols, long-term follow-up, and clinical endpoints—are warranted to establish its safety and efficacy as a next-generation bioactive material for regenerative endodontics.

## Figures and Tables

**Table 1:** Study Characteristics of Included Studies (n = 16).

Author, Year, Study Type	Model/Sample (Species/Cell Type/n)	Intervention (Chitosan-Based: Formulation, Dose/Concentration)	Comparator (Type, Dose)	Outcome Measure (Definition & Unit)	Mean/SD/SE/Event Count	Effect Direction/Trend/Timepoint(s)	Key Findings/Conclusion
Widyastuti et al. 2024 [22] In vivo (rats)	Sprague Dawley rats (n = 18)	Chitosan nanoparticles (CN) from red snapper scales; ionic gelation method	Reversible pulpitis alone; Ca(OH)_2_ application	TGF-β1 expression (ELISA), reparative dentin formation (histopathology)	TGF-β1: *p* = 0.049; Dentin formation: *p* = 0.009	Positive/increased TGF-β1 and dentin formation Not specified	CN enhanced pulp repair and promoted reparative dentin formation compared to control and Ca(OH)_2_
Widyastuti et al. 2024 [15] In vivo (rats)	Sprague-Dawley rats; reversible pulpitis model	Nanochitosan (chitosan nanoparticles) applied as pulp capping	Calcium hydroxide (Ca(OH)_2_); healthy and untreated pulpitis controls	BMP-2 and TGF-β1 levels measured by ELISA (pg/mL)	Significant difference; One-way ANOVA *p* < 0.001 (BMP-2), *p* = 0.016 (TGF-β1)	Positive/Nanochitosan > Ca(OH)_2_ for BMP-2 and TGF-β1 28 days	Nanochitosan increased BMP-2 and TGF-β1 expression more than Ca(OH)_2_ in reversible pulpitis rats
Sularsih et al. 2024 [14] In vivo (rats)	Male Wistar rats (Rattus norvegicus), n = 60, age: 8–16 weeks, weight: 200–250 g	Direct pulp capping with chitosan + hydroxyapatite paste (CH-HA)	KA: glass ionomer cement; KB: Ca(OH)_2_; PA: chitosan; PB: hydroxyapatite	VEGF expression (IHC), number of blood vessels, fibroblast cell proliferation (histology)	Reported as mean ± SD/qualitative comparison (*p* < 0.05)	Positive/increased VEGF, blood vessels, fibroblast proliferation 3, 7, 14 days	CH-HA combination significantly enhanced angiogenesis and fibroblast proliferation compared to all other groups
Hoveizi et al. 2023 [11] In vivo (rats)	Male Wistar rats; rat maxillary left first molar pulp (n not specified)	3D chitosan hydrogel scaffold containing TiO_2_ nanoparticles + human endometrial stem cells (CS/EnSCs/TiO_2_)	Control (no treatment), CS alone, CS + stem cells (CS/SCs)	Dentine formation quality and amount (histological evaluation)	CS/EnSCs/TiO_2_ group showed highest dentine quality and amount; exact numbers not reported	Positive/enhanced dentine formation 8 weeks	Combination of EnSCs + TiO_2_ NPs + chitosan scaffold accelerates and improves dentine regeneration; suitable for direct pulp capping
Zhu et al. 2019 [3] In vitro & in vivo (rats)	Rat model of pulpitis; dental pulp cells in vitro	Silver-doped bioactive glass/chitosan hydrogel (Ag-BG/CS), injectable	Mineral trioxide aggregate (MTA)	Reparative dentin formation, pulp tissue preservation, phosphorylation of p38 and ERK1/2 (MAPK pathway activation)	Reported qualitatively/semi-quantitative	Positive/enhanced reparative dentin formation and pulp preservation Not specified	Ag-BG/CS hydrogel promoted pulp repair and anti-inflammatory effects superior to MTA; enhanced MAPK pathway activation
Huang et al. 2025 [5] In vitro	L929 fibroblast cells (in vitro)	ACS-C bioceramic composite (chitosan + C3S powders; AC20-C formulation)	Not reported	Antibacterial efficacy (% inhibition), cell viability (%), biofilm formation inhibition, inhibition zone, compressive strength	Antibacterial > 90%, cell viability > 80%, clear inhibition zone	Positive/effective Not specified	ACS-C materials show strong antibacterial activity, maintain biocompatibility, and have adequate physical properties for pulp capping
Anaya-Sampayo et al. 2024 [23] In vitro	Human dental pulp stem cells (DPSC) and OB-DPSC (in vitro)	nHA-CH-GEL-PRF scaffold (nano-hydroxyapatite, chitosan, gelatin, platelet-rich fibrin; lyophilized)	nHA-CH-GEL scaffold without PRF	Cell viability (%), cytotoxicity, growth factor release (PDGF-BB, FGF-B)	Not reported numerically	Positive/improved 24 h (growth factor release), unspecified for cell viability	PRF-supplemented scaffolds increase DPSC and OB-DPSC viability; optimal scaffold properties for bone/pulp tissue regeneration
Sornamalar et al. 2024 [16] In vitro	Human dental pulp stem cells (hDPSCs; in vitro)	nPP-CMC scaffold (nano phosphorylated pullulan + carboxymethyl chitosan; 4:5 ratio)	CMC scaffold (group 2), osteogenic medium (group 3)	Cell viability and proliferation (MTT assay, % relative viability)	Significant increase at 21 days vs 7 days (*p* < 0.05); exact numbers not reported	Positive/improved 0, 7, 14, 21 days	nPP-CMC scaffold shows good bioactivity, biocompatibility, and potential for pulp-dentin regeneration
Gould et al. 2024 [10] In vitro	Human dental pulp cells (hDPCs; in vitro)	Chitosan/alginate (C/A) hydrogel with purified bovine pulp and dentin ECM	Hydrogel without ECM (implied control)	Cell proliferation, cytotoxicity, calcium-ion deposition (Alizarin red S), ALP activity, TGF-β expression, chemoattraction	Not reported numerically	Positive/enhanced Not specified	ECM-loaded C/A hydrogels stimulate dental tissue repair, enhance hDPC proliferation, mineralization, ALP activity, and chemoattraction
Kumar et al. 2023 [8] In-vitro	Human dental pulp stem cells (hDPSCs), in vitro	Cobalt-incorporated chitosan (CoCH) scaffold; varying cobalt concentrations, optimal 100 μmol/L in 2% CH, 1:1 ratio	Plain chitosan scaffold	Cytotoxicity (XTT assay), cell adhesion (cell-seeding assay), material characterization (SEM, FTIR, XRD)	Non-cytotoxic; enhanced cell adhesion at optimal Co concentration	Positive/CoCH > CH for cell adhesion Not specified	CoCH scaffold at 100 μmol/L cobalt chloride is biocompatible and enhances hDPSC adhesion, promising for dentin-pulp regeneration
Gurucharan et al.2023 [13] In vitro	Human dental pulp stem cells (hDPSCs; in vitro)	CSHA scaffold: nano-hydroxyapatite + carboxymethyl chitosan (1:5 w/w)	Biodentine	Cell viability/proliferation (MTT), biomineralization (ALP, ARS, OPN), odontogenic/angiogenic markers (DSPP, VEGF)	Cell viability: no significant difference vs Biodentine; ALP, ARS, OPN: higher than Biodentine at 14 days; DSPP/VEGF: upregulated at 21 days	Positive/enhanced differentiation & biomineralization 7, 14, 21 days	CSHA scaffold supports hDPSC viability and proliferation comparable to Biodentine and enhances odontogenic differentiation and mineralization
Ahmed et al. 2023 [1] In vitro	Human dental pulp stem cells (DPSCs; in vitro)	PCL-nano-chitosan scaffold with synthetic hydroxyapatite (PCL-NC-HA) or Mg-substituted hydroxyapatite (PCL-NC-Mg-HA); bioactive materials: MTA, TheraCal LC, Activa Bioactive	Comparison among scaffolds (PCL-NC-HA vs PCL-NC-Mg-HA) and materials (MTA vs TC vs AB)	Odontogenic differentiation (DSPP gene expression), cell viability, proliferation, morphological attachment (SEM, phase contrast)	DSPP expression significantly higher in PCL-NC-Mg-HA; MTA > TC > AB; exact fold changes reported in paper	Positive/enhanced differentiation with PCL-NC-Mg-HA and MTA Multiple timepoints (not specified)	Scaffold composition and MTA most effectively induce odontogenic differentiation of DPSCs
Si Wu et al. 2019 [12] In vitro	Human dental pulp stem cells (DPSCs)	Chitosan/β-glycerophosphate (CS/β-GP) hydrogel delivering VEGF (sustained release)	VEGF alone (without hydrogel)	DPSC proliferation (CCK-8 assay), adhesion, viability, odontogenic differentiation	Reported qualitatively/assay data	Positive/enhanced proliferation and odontogenic differentiation Not specified	CS/β-GP hydrogel allowed sustained VEGF release and enhanced odontogenic differentiation of DPSCs compared to VEGF alone
Bordini et al. 2019 [9] In vitro	Human dental pulp cells (HDPCs)	Porous chitosan–calcium–aluminate scaffold (CH-AlCa) + 1 nM 1α,25-dihydroxyvitamin D3 (1α,25VD)	Plain chitosan scaffold (CH); HDPCs alone	Odontoblastic differentiation markers: ALP activity, mineralized matrix deposition, DSPP/DMP1 mRNA expression, cell migration	Qualitative/relative comparison	Positive/increased odontoblastic differentiation & cell migration Not specified	CH-AlCa scaffold enhances HDPC chemotaxis and odontoblastic differentiation; synergistic effect with low-dose 1α,25VD
Sadeghinia et al. 2019 [7] In vitro	Human dental pulp stem cells (hDPSCs; in vitro)	CS–G/nHA scaffold treated with a-PRP + Fibrin Glue (FG); also FG alone, a-PRP alone, CS–G/nHA alone	CS–G/nHA scaffold alone	Cell adhesion/viability (MTT), osteogenic differentiation (Alizarin red staining, BGLAP, BMP2, RUNX2 expression)	a-PRP–FG/CS–G/nHA group: significantly higher adhesion, mineralization, and osteogenic gene expression; exact numbers not reported	Positive/enhanced 7, 14, 21 days	Composite scaffold treated with a-PRP + FG enhanced adhesion, mineralization, and osteogenic differentiation of h-DPSCs
Hashemi-Beni et al. 2018 [4] In vitro	Stem cells from human exfoliated deciduous teeth (SHED; in vitro; n not specified)	PHB/chitosan/nano-bioglass (nBG) scaffold; also PHB, PHB/chitosan, PHB/chitosan/nBG + MTA	PHB scaffold, PHB/chitosan scaffold, MTA alone	Cell viability/proliferation (MTT assay)	PHB/chitosan/nBG scaffold and PHB/chitosan/nBG + MTA showed significantly higher viability at day 7; exact numbers not reported	Positive/enhanced viability 3, 5, 7 days	Scaffolds containing nBG nanoparticles are more biocompatible and promote SHED proliferation better than other scaffolds

Abbreviations: CN, chitosan nanoparticles; CH, chitosan; HA, hydroxyapatite; nHA, nano-hydroxyapatite; PRF, platelet-rich fibrin; a-PRP, activated platelet-rich plasma; FG, fibrin glue; ECM, extracellular matrix; PCL, polycaprolactone; PHB, polyhydroxybutyrate; CMC, carboxymethyl chitosan; CoCH, cobalt-incorporated chitosan; TiO_2_, titanium dioxide; MTA, mineral trioxide aggregate; Ca(OH)_2_, calcium hydroxide; VEGF, vascular endothelial growth factor; BMP-2, bone morphogenetic protein-2; TGF-β_1_, transforming growth factor beta-1; DSPP, dentin sialophosphoprotein; ALP, alkaline phosphatase; OPN, osteopontin; ARS, alizarin red staining; DMP1, dentin matrix protein 1; OD, optical density; SD, standard deviation; SE, standard error.

**Table 2:** SYRCLE Risk of Bias Summary (In Vivo Studies) [17].

Study	1	2	3	4	5	6	7	8	9	10	Overall
Widyastuti et al. 2024 [22]	U	L	U	U	H	U	H	L	L	U	H
Widyastuti et al. 2024 [15]	U	L	U	U	H	U	U	L	L	U	U
Sularsih et al. 2024 [17]	U	L	U	U	H	U	U	L	L	U	U
Hoveizi et al. 2023 [11]	U	L	U	U	H	U	U	L	L	U	U
Zhu et al. 2019 [3]	U	U	U	U	U	U	U	L	L	U	H

Abbreviations: L = Low risk; U = Unclear risk; H = High risk. Domains: (1) Sequence generation, (2) Baseline characteristics, (3) Allocation concealment, (4) Random housing, (5) Blinding of caregivers/investigators, (6) Random outcome assessment, (7) Blinding of outcome assessor, (8) Incomplete outcome data, (9) Selective outcome reporting, (10) Other sources of bias.

**Table 3:** Risk of Bias Assessment for Quantitatively Analyzed In Vitro Studies [18].

Study (Author, Year)	1	2	3	4	5	6	7	8	9	10	Overall
Sornamalar et al. 2024 [16]	L	L	L	U	H	L	L	L	L	U	L
Gurucharan et al. 2022 [13]	L	L	L	U	U	L	L	L	L	U	M
Sadeghinia et al. 2019 [7]	L	L	L	U	H	L	L	L	L	U	L
Wu et al. 2019 [12]	L	L	L	U	H	L	L	L	L	U	L
Hashemi et al. 2018 [4]	L	L	L	U	H	L	L	L	L	U	L

Abbreviations: L = Low risk, U = Unclear, H = High risk; Overall risk interpreted as: Low (≥16 points), Moderate (10–15), High (<10). Domains: (1) Test System Described, (2) Control Adequacy, (3) Replication, (4) Randomization of Treatment, (5) Blinding of Measurement, (6) Assay Validation/Calibration, (7) Data Completeness, (8) Outcome Reporting, (9) Statistical Analysis, (10) Funding/COI Transparency.

**Table 4:** Direction-of-Effect Coding and Weighting Log.

Study (Author, Year)	Model Type	Outcome Domain(s)	Comparator Group	Effect Direction (+1 = Positive, 0 = Neutral, –1 = Negative)	Evidence Weight (2 = Mean ± SD; 1 = p-Only; 0.5 = Qualitative)	Primary Finding Summary	Rationale for Code
Widyastuti et al. 2024 [15]	In vivo	BMP-2, TGF-β_1_ (pg/mL)	Ca(OH)_2_	1	2	Nanochitosan ↑ BMP-2 & TGF-β_1_ vs Ca(OH)_2_ (*p* < 0.001)	Numeric ELISA data with ANOVA significance
Widyastuti et al. 2024 [22]	In vivo	TGF-β_1_ (pg/mL), Reparative dentin	Ca(OH)_2_/control	1	2	CN ↑ TGF-β_1_ expression and dentin formation (*p* < 0.05)	ELISA numeric data provided
Sularsih et al. 2024 [14]	In vivo	VEGF (IHC), Fibroblast proliferation	Ca(OH)_2_/GIC/HA alone	1	1.5	CH-HA ↑ angiogenesis & fibroblast count (*p* < 0.05)	Means ± SD reported; semi-quantitative IHC
Hoveizi et al. 2023 [11]	In vivo	Dentin formation (histologic score)	CS alone/no treatment	1	1	CS/EnSCs/TiO_2_ ↑ dentin quality and amount	Descriptive with qualitative scoring
Zhu et al. 2019 [3]	In vivo + in vitro	Reparative dentin, Inflammation/MAPK activation	MTA	1	1	Ag-BG/CS > MTA for pulp repair & anti-inflammatory effect	Qualitative histology and western blot
Sornamalar et al. 2024 [16]	In vitro	Cell viability (MTT %)	CMC scaffold	1	2	nPP–CMC ↑ relative viability vs CMC (*p* < 0.05)	Table data numeric and significant
Gurucharan et al. 2022 [13]	In vitro	Cell viability (MTT OD 570 nm), ALP, OPN expression	Biodentine	1	2	CSHA scaffold ↑ differentiation & biomineralization (*p* <0.05)	Multiple markers improved vs comparator
Wu et al. 2019 [12]	In vitro	Cell proliferation (CCK-8 OD 450 nm), VEGF release	NC (no hydrogel)	1	2	CS/β-GP hydrogel ↑ proliferation and VEGF sustained release	Quantitative mean ± SD visible on figure
Sadeghinia et al. 2019 [7]	In vitro	Cell viability (MTT %)/Mineralization	Control	1	2	a-PRP–FG/CS–G/nHA ↑ adhesion & viability (*p* < 0.0001)	Clear quantitative difference; replicates = 3
Hashemi-Beni et al. 2018 [4]	In vitro	Cell viability (MTT OD 540 nm)	PHB scaffold/control	1	2	PHB/CH/nBG + MTA ↑ cell viability vs control (*p* < 0.05)	Quantitative mean ± SD reported; consistent significance

Abbreviations: CN = chitosan nanoparticles; CH-HA = chitosan–hydroxyapatite; CS/EnSCs/TiO_2_ = chitosan scaffold + endometrial stem cells + titanium dioxide; Ag-BG/CS = silver-doped bioactive glass/chitosan hydrogel; nPP–CMC = nano-phosphorylated pullulan/carboxymethyl chitosan; CSHA = carboxymethyl chitosan + nano-hydroxyapatite scaffold; CS/β-GP = chitosan/β-glycerophosphate hydrogel; a-PRP–FG/CS–G/nHA = activated platelet-rich plasma + fibrin glue + chitosan/gelatin/nano-hydroxyapatite scaffold; PHB/CH/nBG = polyhydroxybutyrate/chitosan/nano-bioglass scaffold; Ca(OH)_2_ = calcium hydroxide; MTA = mineral trioxide aggregate; GIC = glass ionomer cement; BMP-2 = bone morphogenetic protein-2; TGF-β_1_ = transforming growth factor beta-1; VEGF = vascular endothelial growth factor; ALP = alkaline phosphatase; OPN = osteopontin; SD = standard deviation; OD = optical density; ↑= increased.

**Table 5:** GRADE-Preclinical certainty of evidence for the biological efficacy of chitosan-based nanoparticles and biomaterials in pulp capping and regeneration [20].

Certainty-Assessment Criteria	Evaluation		
Number of studies	5 animal RCTs (≈253 samples) + 11 in vitro studies		
Study design	Controlled preclinical experiments (no human data)		
Risk of bias	Serious—randomization and blinding rarely reported		
Inconsistency	Serious—variation in formulations, models, and assays		
Indirectness	Very serious—animal and in vitro evidence only; surrogate endpoints		
Imprecision	Serious—small sample sizes and incomplete variance data		
Other considerations	Publication bias likely—all included studies reported positive effects		
Outcome	Comparator	Relative Effect (95% CI)	Absolute Effect (95% CI)
Pulp healing and reparative dentin formation with chitosan-based nanomaterials	Conventional pulp-capping agents (Ca(OH)_2_, MTA, GIC)	Not estimable—no pooled quantitative data	Descriptive synthesis only
Overall certainty of evidence (GRADE-Preclinical)		⨁⨁◯◯ Low to Very Low	
Importance		Biologically promising preclinical results but limited translational certainty for human use	

## Data Availability

Data will be available from corresponding upon reasonable request.

## Supplementary Materials

The following supporting information can be downloaded at: https://www.mdpi.com/article/10.3390/biomimetics10120822/s1, Table S1: Excluded studies with reason, Figure S1: Bubble plot, Figure S2: Heatmap.
