# Supplementary material for: Chitosan-Based Nanoparticles and Biomaterials for Pulp Capping and Regeneration: A Systematic Review with Quantitative and Evidence-Mapping Synthesis
Source: Biomimetics (Basel). 2025 Dec 9;10(12):822. doi: 10.3390/biomimetics10120822 (PMC12730371; doi:10.3390/biomimetics10120822)
Supplement: Supplementary file 1 [file biomimetics-10-00822-s001.zip › Table S1.pdf]

Sr. No	Article	Reason
1.	Cytotoxic effect of chitosan nanoparticles on normal human dental pulp cells	Toxicity effect of chitosan
2.	Evaluation of dental pulp stem cells behavior after odontogenic differentiation induction by three different bioactive materials on two different scaffolds	Chitosan not studied
3.	Genotoxicity Induced by Cellular Uptake of Chitosan Nanoparticles in Human Dental Pulp Cells	Toxicity effect of chitosan
4.	Influence of photodynamic therapy on bond strength and adhesive interface morphology of MTA based root canal sealer to different thirds of intraradicular dentin	Shear bond strength
5.	Nanoparticles and Their Antibacterial Application in Endodontics	Review article
6.	Antibacterial Properties Associated with Chitosan Nanoparticle Treatment on Root Dentin and 2 Types of Endodontic Sealers	Endodontic sealer
7.	Antibacterial Properties of Chitosan Nanoparticles and Propolis Associated with Calcium Hydroxide against Single- and Multispecies Biofilms: An In Vitro and In Situ Study	Biofilm development study
8.	Chitosan-Based Biomimetically Mineralized Composite Materials in Human Hard Tissue Repair	Review article
9.	Cell viability and apoptotic changes of dental pulp stem cells treated with propolis, chitosan, and their nano counterparts	Review article
10.	Evaluation of Dentinogenisis of Bionanocomposite Scaffold versus Mineral Trioxide Aggregate as A Direct Pulp Capping Material for Vital Pulp Therapy: An Animal Study	Different types of biocompatible material
11.	Comparative evaluation of bioactivity of MTA plus and MTA plus chitosan conjugate in phosphate buffer saline an invitro study	MTA based study
12.	Effect of root dentin conditioning using different chelating agents on pushout bond strength of MTA-fillapex and bioroot RCS: An in vitro study	Sealer study
13.	Antimicrobial activity of endodontic sealers and medications containing chitosan and silver nanoparticles against Enterococcus faecalis	Endodontic sealer study
14.	Investigation of a Novel Injectable Chitosan Oligosaccharide— Bovine Hydroxyapatite Hybrid Dental	Bio-composite study

	Biocomposite for the Purposes of Conservative Pulp Therapy	
15.	To Evaluate and Compare the Microleakage of Chitosan Nano Hydrogel-Incorporated MTA and Glass Ionomer Cements When used as a Retrograde Filling Material	Microleakage study

Supplementary Table S1: Excluded studies with reason
