# Supplementary figures and images for: Chitosan-Based Nanoparticles and Biomaterials for Pulp Capping and Regeneration: A Systematic Review with Quantitative and Evidence-Mapping Synthesis
Source: Biomimetics (Basel). 2025 Dec 9;10(12):822. doi: 10.3390/biomimetics10120822 (PMC12730371; doi:10.3390/biomimetics10120822)

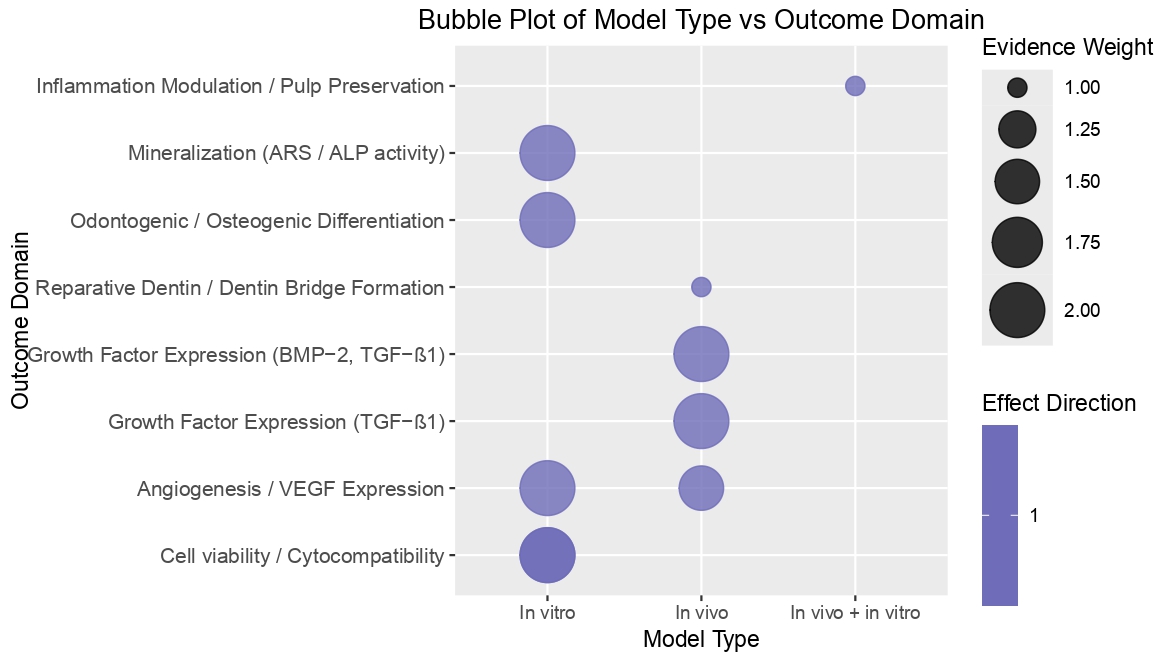

Supplement: Supplementary file 1 [file biomimetics-10-00822-s001.zip › Figure S1.jpg]

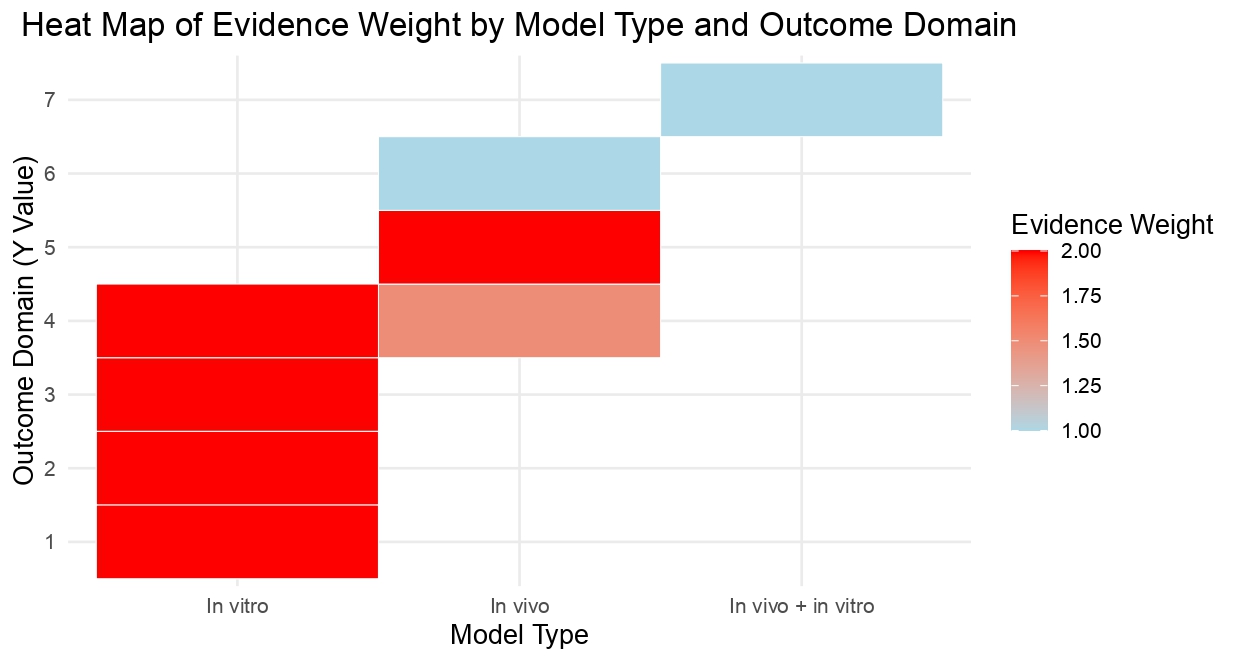

Supplement: Supplementary file 1 [file biomimetics-10-00822-s001.zip › Figure S2.jpg]
